# Interaction of *Sesbania mosaic virus* (SeMV) RNA-dependent RNA polymerase (RdRp) with the p10 domain of polyprotein 2a and its implications in SeMV replication

**DOI:** 10.1016/j.fob.2014.03.009

**Published:** 2014-03-29

**Authors:** Kunduri Govind, Arindam Bakshi, Handanahal S. Savithri

**Affiliations:** Department of Biochemistry, Indian Institute of Science, Bangalore 560012, India

**Keywords:** Sobemovirus, Sesbania mosaic virus, Replication, RNA-dependent RNA polymerase (RdRp), Protein-protein interactions, 3AT, 3 amino-1,2,4 triazol, CP, coat protein, IPTG, isopropyl-1thio-β-d-galactopyranoside, LacZ, β-galactosidase, LB, Luria Bertani broth, MEL1, α-galactosidase, MP, movement protein, Ni–NTA, nickel–nitrilo tri-acetic acid, ONPG, ortho-nitrophenyl-β-galactoside, PBST, phosphate buffered saline with 0.1% TWEEN 20, Pro, protease, RdRp, RNA-dependent RNA polymerase, SD, synthetic dropout, *SeMV*, *Sesbania mosaic virus*, VPg, viral protein genome linked, Y2H, yeast two hybrid

## Abstract

Identification of viral encoded proteins that interact with RNA-dependent RNA polymerase (RdRp) is an important step towards unraveling the mechanism of replication. *Sesbania mosaic virus* (SeMV) RdRp was shown to interact strongly with p10 domain of polyprotein 2a and moderately with the protease domain. Mutational analysis suggested that the C-terminal disordered domain of RdRp is involved in the interaction with p10. Coexpression of full length RdRp and p10 resulted in formation of RdRp–p10 complex which showed significantly higher polymerase activity than RdRp alone. Interestingly, CΔ43 RdRp also showed a similar increase in activity. Thus, p10 acts as a positive regulator of RdRp by interacting with the C-terminal disordered domain of RdRp.

## Highlights

• SeMV RdRp strongly interacts with p10 domain of polyprotein 2a.
• C-terminal disordered domain of RdRp is required for interaction with p10.
• p10 acts as a positive regulator of RdRp activity.

## 1 Introduction

The replication cycle of a positive stranded RNA virus is a complex event involving the synthesis of the negative strand followed by asymmetric synthesis of the progeny positive strands. Replicase is a key enzyme consisting of host and viral encoded proteins that catalyzes this complete replication *in vivo* [1–3]. Bacteriophage Qβ replicase contains virus encoded RNA-dependent RNA polymerase (RdRp) (β subunit) and three host proteins namely elongation factor thermo unstable (EF-Tu), elongation factor thermo stable (EF-Ts) and ribosomal protein S1 [4–6]. Similarly, highly purified replication complex of *Tomato bushy stunt virus* (TBSV) was shown to contain two viral encoded proteins (p33 and p92^pol^) and four host proteins including heat shock protein 70 (HSP 70 or Ssa1/2p in yeast), glyceroldehyde 3 phosphate dehydrogenase (GAPDH or Tdh2/3p in yeast), pyruvate decarboxylase (Pdc1p) and an unidentified acidic protein [7].

In many viruses, several viral encoded proteins are able to interact directly with RdRp domain and modulate the polymerase activity. For example, Hepatitis C-virus (HCV) NS5A interacts with NS5B (RdRp) and modulates the polymerase activity [8]. Further, the NS3 (helicase/proteinase) and NS4B proteins also physically interact with NS5B and positively and negatively regulate the polymerase activity respectively [9]. The 3Dpol (RdRp) of poliovirus was shown to interact with the 3C dimer (two molecules of 3CD protease) and facilitate uridylylation of VPg [10]. *Alfalfa Mosaic Virus* (AMV) or *Ilarviruses* coat protein (CP) was shown to be an integral component of replicase [11] that binds to 3′ end of the genomic RNA and thereby position the RdRp at the initiation site for the minus strand synthesis [12]. The p33 protein of TBSV, 126 K protein of *Tobacco mosaic virus* (TMV) and 1a protein of *Brome mosaic virus* (BMV) and P1 protein of AMV were shown to be part of their respective replication complexes [13].

*Sesbania mosaic virus* (SeMV) is a single stranded positive sense RNA virus that belongs to the genus *Sobemovirus*. The viruses from this genus infect mono or dicotyledonous plants. The viral genome is compact and encodes for 3 overlapping reading frames. The 5′ and 3′ proximal open reading frames (ORF) encode for movement protein (MP) and coat protein (CP) respectively [14]. The central ORF encodes for two polyproteins 2a and 2ab [15] and they have a domain arrangement of Membrane anchor-Protease (pro) – viral protein genome linked (VPg) – p10 – p8 and Membrane anchor-pro-VPg-RdRp respectively [15,16]. The RdRp domain is expressed via the −1 ribosomal frame shifting mechanism [17].

*In vitro* studies with SeMV recombinant RdRp showed that it could synthesize RNA in a primer independent manner and produce double stranded RNAs as end product [18]. Towards understanding of complete replication mechanism observed *in vivo*, we have attempted to identify the virus encoded ancillary proteins that interact with RdRp. Using a yeast two hybrid (Y2H) system the RdRp was found to interact strongly with the p10 domain and moderately with the protease domain. Further biochemical studies indicated that the p10 interacts with C-terminal domain of RdRp and positively regulates the RdRp activity.

## 2 Materials and methods

### 2.1 Cloning

The gene sequences corresponding to Δ70pro, VPg, p10, p8, RdRp and CP were PCR amplified using appropriate forward and reverse primers (Table 1) and high fidelity Phusion polymerase. The SeMV full length cDNA (pFX37) clone [19,20] was used as a template in PCR reactions. The PCR products of ΔN70pro, VPg and CP were cloned at SmaI site of pGBKT7 vector. The PCR products of p10 and p8 were cloned at NdeI and BamHI site of pGBKT7 vector. The RdRp PCR product was cloned at SmaI site of pGADT7 vector.

The deletion mutants of RdRp namely CΔ43 and CΔ85 were obtained by cloning the corresponding PCR products (generated by using the appropriate forward and reverse primers listed in Table 1) at SmaI site of pGADT7. The mutant clones were designated as pGADT7-RdRp CΔ43 and pGADT7-RdRp CΔ85 respectively.

The pRSF duet p10-RdRp and RdRp deletion mutants (p10-RdRpCΔ43 and p10-RdRpCΔ85) were constructed by initial cloning of p10 gene fragment at EcoRI and SalI sites of first multiple cloning site (gives N-terminal His tag to p10) followed by cloning of RdRp and its mutant PCR products at NdeI & XhoI sites of pRSF duet vector second multiple cloning site (RdRp lacks His Tag).

The pRSET clones ΔN70 Pro, VPg and p8 were kind gifts from Dr. Nair [16,21]. The untagged pRSF duet RdRp was cloned at NdeI & EcoRV sites.

The PCR products of RdRp and RdRp CΔ43 were cloned at NdeI & XhoI sites of pET 22b vector. The expressed proteins contain C-terminal His tag.

### 2.2 Yeast two hybrid (Y2H) interaction assay

The AH109 yeast strain was cotransformed with appropriate pGBKT7 and pGADT7 clones and plated on leucine^−^ (L^−^) and tryptophan^−^ (W^−^) synthetic dropout (SD) plates. The procedure used for transformation and preparation of plates were from yeast protocols handbook [PT3022-1(PR13103) Clontech]. The AH109 strain contains HIS3, ADE2, MEL1 and lacZ reporter constructs that allows high stringency Y2H selection. The SD medium contained 0.67% yeast nitrogen base, 2% glucose or 2% galactose & 1% Raffinose and lacked appropriate amino acids used for selection. For preparation of plates, 2% agar was also added before autoclaving. The colonies from L^−^ and W^−^ plates were initially re-streaked on fresh L^−^ and W^−^ plates and subsequently used for replica plating. The cells from L^−^ & W^−^ plates were replica plated sequentially with increasing stringency of selection and analyzed for growth on L^−^, W^−^ & histidine^−^ (H^−^) (triple dropout)/L^−^, W^−^ & H^−^ containing 3 amino-1,2,4 triazol (3AT) (triple dropout +3AT)/L^−^, W^−^, H^−^ and Adenine^−^ (Ade^−^) (quadruple dropout). The plates were incubated at 30 °C for 3–4 days.

### 2.3 β-Galactosidase assay

The β-galactosidase assay was carried out to validate and to determine the strength of protein–protein interactions. The ortho-nitrophenyl-β-galactopyranoside (ONPG) was used as a substrate. The cells were inoculated into L^−^ and W^−^ media (for transformants which did not grow on quadruple dropout plates) or L^−^, W^−^, H^−^ and Ade^−^ (for those which showed positive growth on quadruple dropout plate) media and grown at 28 °C with vigorous shaking until the OD_600_ reached 0.6–1. Cells (1 ml) were pelleted and lysed with glass beads and β-galactosidase assay was carried out as described in yeast protocols handbook [PT3022-1(PR13103) Clontech]. The β-galactosidase activity was estimated using the following formula 1000 × (OD_420_/*t* × *V* × OD_600_), wherein OD_420_ represents the product absorbance, OD_600_ cell density, *t*, time of incubation in min and *V*, volume of cells in ml.

### 2.4 Expression of RdRp and its C-terminal deletion mutants in BL21(DE3)pGro7 cells

BL21 (DE3) pGro7 competent cells were transformed with pET-22b RdRp or its mutants and plated on LB agar plates containing Ampicillin (amp) (50 μg/ml) and Chloramphenicol (chl) (25 μg/ml). A single colony was inoculated to 20 ml LB containing 50 μg/ml Amp, 25 μg/ml Chl and grown overnight. This culture was inoculated to 500 ml of 2 × LB containing the antibiotics and allowed to grow till the OD_600_ reached 0.4, following this 0.5 mg/ml l-arabinose was added and allowed to grow further until OD_600_ reached to 0.6–0.8. The culture at this stage was stored at 4 °C for 1 h and induced with 0.3 mM IPTG for 6 h at 15 °C. The cells were harvested by centrifugation and resuspended in 40 ml of lysis buffer (20 mM Tris–HCl pH 7.5, 5 mM MgCl_2_, 400 mM NaCl, 10 mM imidazole, 0.1 mM DTT and 0.1% non-ionic detergent p40). Resuspended cells were sonicated for 20–30 min at amplitude of 30 (Vibra cell) and the lysate was centrifuged at 10,000*g* (Avanti JE, Beckmen coulter) for 10 min at 4 °C. The supernatant fraction of the lysate was incubated with 1 ml of Nickel–Nitrilo tri-acetic acid (Ni–NTA) (Novagen) beads for 2–3 h at 4 °C. The beads were packed into a column and washed with 100 ml of lysis buffer, 50 ml of lysis buffer containing 1 mM ATP and 100 mM KCl, followed by washing with 50 ml of 20 mM imidazole (in lysis buffer) and finally the protein was eluted with lysis buffer containing 300 mM imidazole. The eluted fractions were analyzed by SDS–PAGE. The fractions containing purified protein were pooled and dialyzed against 20 mM Tris–HCl buffer pH 7.5, containing 5 mM MgCl_2_ and 0.1 mM DTT.

### 2.5 Coexpression of RdRp and its deletion mutant with p10

One of the coexpressed proteins contains His tag (p10 in duet clones) and the other protein lacks tag (RdRp and its mutant). The pRSF duet clone containing p10 and RdRp full length or RdRp C-terminal deletion mutant were transformed into BL21 (DE3) pGro7 *Escherichia coli* cells and proteins were coexpressed as described above. The cells were harvested by centrifugation at 10,000*g* and resuspended in 2 ml of PBST buffer (phosphate buffered saline with 0.1% Tween 20) containing 5 mM β-mercapto ethanol (lysis buffer). The cells were lysed by sonication and the debris was removed by centrifugation at 10,000*g*. To the supernatant Ni–NTA beads were added and left on end to end rotor for 3 h. Beads were collected and washed with lysis buffer containing 20 mM imidazole. The proteins were eluted with lysis buffer containing 300 mM imidazole. The eluted proteins were analyzed by SDS–PAGE and western blotting.

### 2.6 Pull down assays for protein–protein interactions

The protein–protein interactions observed using Y2H assay were verified using hexa Histidine tag (His) based pull down method. The His tagged proteins and untagged proteins were expressed independently in *E. coli* (in100 ml of LB). The cells were pelleted by centrifugation at 10,000*g* and resuspended in 2 ml of PBST buffer. The cells expressing untagged protein (1 ml) was mixed with the cells expressing His tagged protein (1 ml) and the volume was adjusted to 10 ml with PBST buffer. The cells were lysed by sonication, and the debris was removed by centrifugation at 10,000*g*. The supernatant was incubated on ice for one hour, followed by the addition of 200 μl of Ni–NTA beads and left on end to end rotor for 3 h. Beads were collected and washed with PBST and PBST with 20 mM immidazole (3 times each with 10 ml). The proteins were eluted with 1 ml of elution buffer (PBST containing 300 mM imidazole). The eluted proteins were analyzed by SDS–PAGE and western blotting.

### 2.7 *In vitro* RdRp assay

The RdRp assay was carried out essentially as described earlier [18]. Briefly, 2 μg of RdRp and the deletion mutant with and without p10 was used to carry out the RdRp assay in presence of αP^32^ UTP at 30 °C for 1 h. The reaction was stopped by addition of 10 mM EDTA. The amount of radioactivity was measured by filter binding assay [18].

## 3 Results

### 3.1 Identification of cognate viral proteins that interact with RdRp

**Fig. 1:**
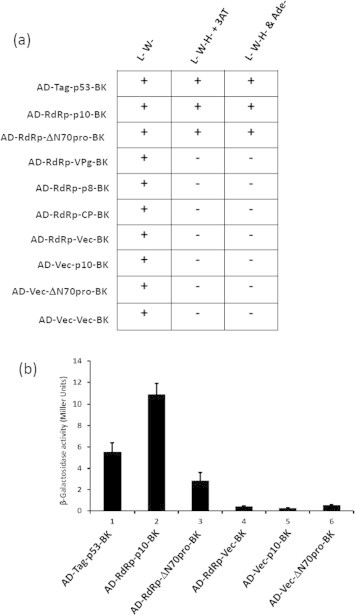
**(**a) Y2H assay to detect ancillary proteins that interact with RdRp. The pGADT7 RdRp was cotransformed in pairs with one of the following clones of pGBK p10/ΔN70pro/VPg/p8 or CP or empty vector into AH109 strain and plated on L, W SD transformation selection plates. The pGAD-RdRp-Vector-pGBK, pGAD-vec-p10-pGBK, pGAD-Vector-ΔN70pro-pGBK and pGAD-Vec-Vec-pGBK were used as negative controls. The pGAD-T antigen-p53-pGBK cotransformant was used as positive control. The summary of Y2H screen results is presented as a table. (b) β-Galactosidase assay to quantitate the strength of interactions: The cotransforments which showed positive interaction were inoculated into quadruple dropout media and grown at 28 °C until the OD_600_ reached to 0.6–1. The assay was performed and β-galactosidase activity units were calculated as described in Section 2. The data is presented as a bar diagram (*Y*-axis, β-galactosidase activity units (miller units); *X*-axis Y2H interactions). The pGAD-RdRp-Vector-pGBK, pGAD-Vector-ΔN70pro-pGBK and pGAD-Vector-p10-pGBK (grown in L, W galactose carbon source) were used as negative control. The pGAD-T-antigen-pGBKp53 was used as positive control.

The pGADT7-RdRp was cotransformed with pGBKT7 clones harboring ΔN70 pro, VPg, p10, p8 or CP constructs separately and plated on L^−^ & W^−^ SD plates. The AH109 cotransformed with pGBKT7-p53 (Murine p53 fused to Gal4 DNA binding domain) and pGADT7-T antigen (SV40 large T-antigen fused to Gal4 activation domain) was used as positive control. The AH109 cotransformed with pGADT7-RdRp and pGBKT7 vector; pGADT7-Vec and pGBKT7-p10 and pGADT7 vector-pGBKT7 vector; pGADT7Vector and pGBKT7pro were used as negative controls (Fig. 1a). All the cotransformation experiments were performed three times and the results obtained are summarized in Fig. 1a.

Appearance of colonies on L^−^, W^−^ plates indicates that the double transformation has occurred (pGBKT7 vector contains the TRP1 coding sequence and pGADT7 vector contains the LEU2 coding sequence). The colonies which appeared on L^−^ and W^−^ plates were re-streaked onto fresh L^−^ and W^−^ plates for use in further replica plating experiments (Fig. 1a column 1, L^−^, W^−^). The colonies from L^−^ and W^−^ plates were re-streaked on L^−^, W^−^ and H^−^ plates (triple drop out or medium stringency) to select positive interactions. However, growth was observed in all the cotransformations including negative control (pGADT7 RdRp and pGBKT7 Vec). The growth on H plates could be due to auto-activation or leaky expression of His gene. In order to inhibit leaky expression of His gene and to identify positive interactions, colonies obtained on triple dropout were re-streaked on triple dropout plates containing 5 mM 3AT (3 amino-1,2,4 triazol). The 3AT is a competitive inhibitor of yeast His3 protein (His3p) and was used to inhibit low levels of expression of His3p, thereby suppressing the background growth [22,23]. In the presence of 3AT, as expected negative control did not grow and at the same time the positive control could grow suggesting 3AT completely inhibited the leaky expression of His gene (Fig. 1a, L^−^, W^−^, H^−^ 5 mM 3AT, column 2). Interestingly, colonies were observed when RdRp-ΔN70pro and RdRp-p10 cotransformants were streaked on triple dropout plates containing 3AT (L^−^, W^−^, H^−^ + 3AT) and quadruple dropout plates (L^−^, W^−^, H^−^ and Ade^−^) plates (high stringency) suggesting that RdRp-p10 and RdRp-Δ70pro might interact (Fig. 1a, L^−^, W^−^, H^−^ and Ade^−^ (column 3)). However, there was no growth observed when RdRp was cotransformed with VPg, p8 and CP expression constructs suggesting that these proteins might not interact strongly (Fig. 1a).

In order to validate and quantitate the relative binding strengths of these interactions, a β-galactosidase assay was carried out using ONPG substrate. The RdRp-ΔN70pro and RdRp-p10 cotransformants were inoculated into L^−^, W^−^, H^−^ and Ade^−^ medium and grown at 28 °C until the OD_600_ reached to 0.6–1. The cells were pelleted and β-galactosidase assay was performed as described in the Section 2. The activity levels were compared with positive control in which AH109 cells were cotransformed with pGBKT7-p53 (Murine) and pGADT7-SV40 large T-antigen and the negative controls in which pGADT7-RdRp and pGBKT7-vector or pGADT7-Vec and pGBKT7-p10 or pGADT7 vector and pGBKT7pro were cotransformed (negative controls were grown in L^−^, W^−^ galactose media). As shown in Fig. 1b, the product formation upon coexpression of RdRp-p10 (bar 2) cotransformants was even higher than the positive control (bar 1), suggesting a strong interaction. However, a significant amount of product was also formed when the assay was performed with lysates from cells cotransformed with RdRp-ΔN70pro (bar 3). These results suggested that SeMV RdRp could interact strongly with protein p10 and moderately with protease domain of polyprotein 2a. Due to strong interaction observed between RdRp and p10, further experiments were conducted to delineate the importance of such an interaction.

### 3.2 *In vitro* pull down assays

**Fig. 2:**
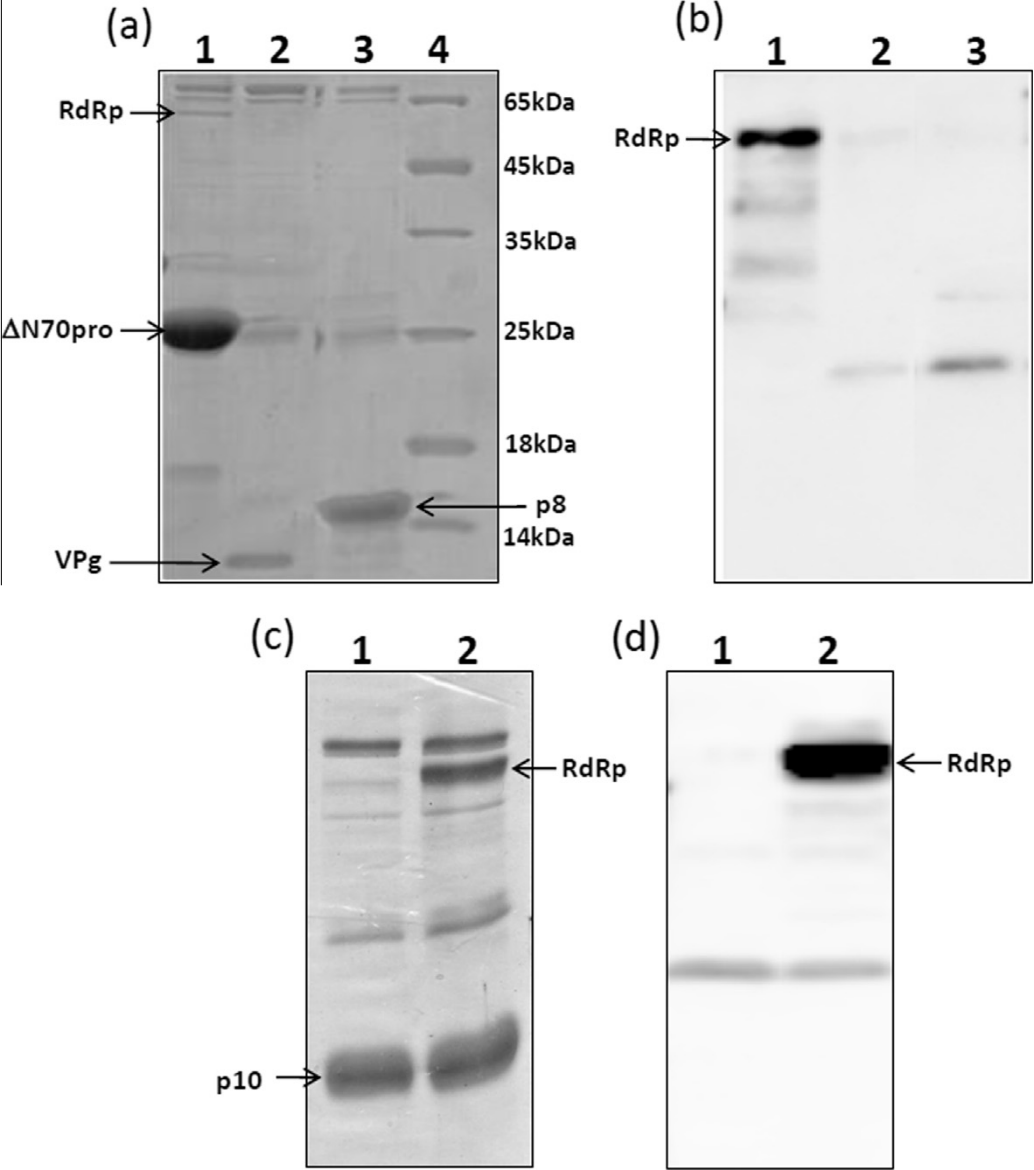
Pull down assays for protein–protein interactions: Hexa Histidine tag based pull down assays were carried out to demonstrate protein-protein interactions *in vitro*. (a) SDS PAGE analysis of samples stained with commassie blue Lanes 1–3 shows pull down of 6x His tagged ΔN70 Pro, VPg, and p8 after incubation with untagged RdRp respectively. (b) Western blot analysis of respective samples from (a) with anti RdRp antibodies. (c) Coexpression of 6x His-p10 and untagged RdRp followed by pull down of 6x His tag p10 with Ni–NTA beads. Lane 1, p10 alone; lane 2 6x His-p10 coexpressed with RdRp which does not contain His tag. (d) Western blot analysis of samples from (c) with anti RdRp antibodies.

To further validate the interactions observed with Y2H system, a series of pull down experiments were carried out. The RdRp-ΔN70pro, RdRp-VPg and RdRp-p8 interactions were verified by incubating lysates of His tagged ΔN70Pro, VPg, and p8 with untagged RdRp and pull down with Ni–NTA beads as described in the Section 2. The eluted fractions were analyzed by SDS–PAGE and western blotting with anti RdRp antibodies (Fig. 2(a,b)). Consistent withY2H results, western blot analysis indeed detected RdRp only when pull down was carried out with ΔN70pro but not with VPg and p8 Fig. 2(a,b). However amount of RdRp bound to protease was low, probably due to weak interaction. An *E. coli* protein with a molecular weight of about 65–70 kDa was observed in all the pull downs however identity of this protein is not clear (Fig. 2(a)). Similarly, no significant interaction was observed between RdRp and CP (data not shown).

To test interaction between RdRp and p10 a coexpression approach was taken, wherein, RdRp and p10 were cloned in a single pRSF Duet vector and coexpressed in BL21 DE3 *E. coli* cells expressing chaperones GroEL and GroES. It was observed that solubility of full length RdRp improved significantly when coexpressed with chaperones (data not shown). The p10 contains His tag at the N-terminus and RdRp lacks His tag. The RdRp was pulled down with p10 using Ni–NTA affinity beads, washed and analyzed by SDS–PAGE and western blotting. As shown in Fig. 2(c and d), a significant amount of RdRp copurified along with p10, suggesting that these two proteins interact strongly.

### 3.3 Identification of p10 interacting regions on RdRp

**Supplementary Fig. 1:**
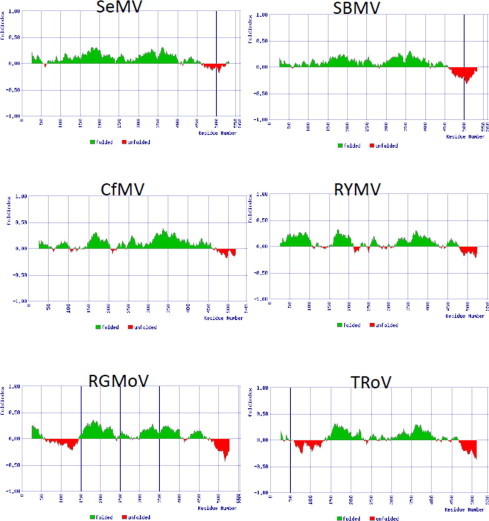
Fold index analysis of RdRp domains of *Sobemovirus*. The unfolded regions in RdRp domains were predicted using fold index program with default values. The output file obtained after fold index analysis is shown. The unfolded region is shown in red color with negative values. The C-terminal unfolded region is present in all members of *Sobemovirus* including Southern bean mosaic virus (SBMV); Lucerne transient streak virus (LTSV); Cocksfoot mottle virus (CfMV); Rice yellow mottle virus (RYMV); Ryegrass mottle virus (RGMoV); Turnip rosette virus (TRoV) RdRps.

**Fig. 3:**
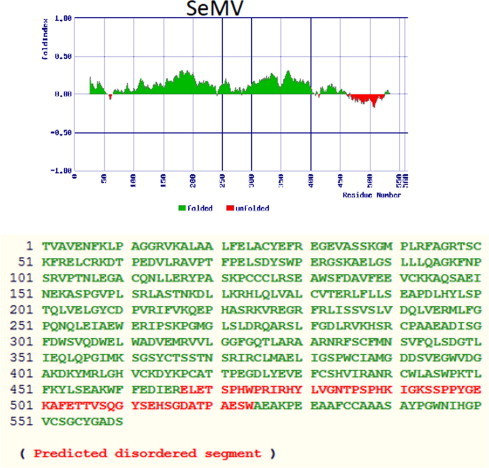
In silico analysis of RdRp: The unfolded regions in RdRp domain was predicted using fold index program with default values. The output file obtained after fold index analysis is shown. The unfolded region is shown in red with negative values. The predicted disordered segment is also shown in red within the amino acid sequence of SeMV RdRp. (For interpretation of the references to colour in this figure legend, the reader is referred to the web version of this article.)

**Fig. 4:**
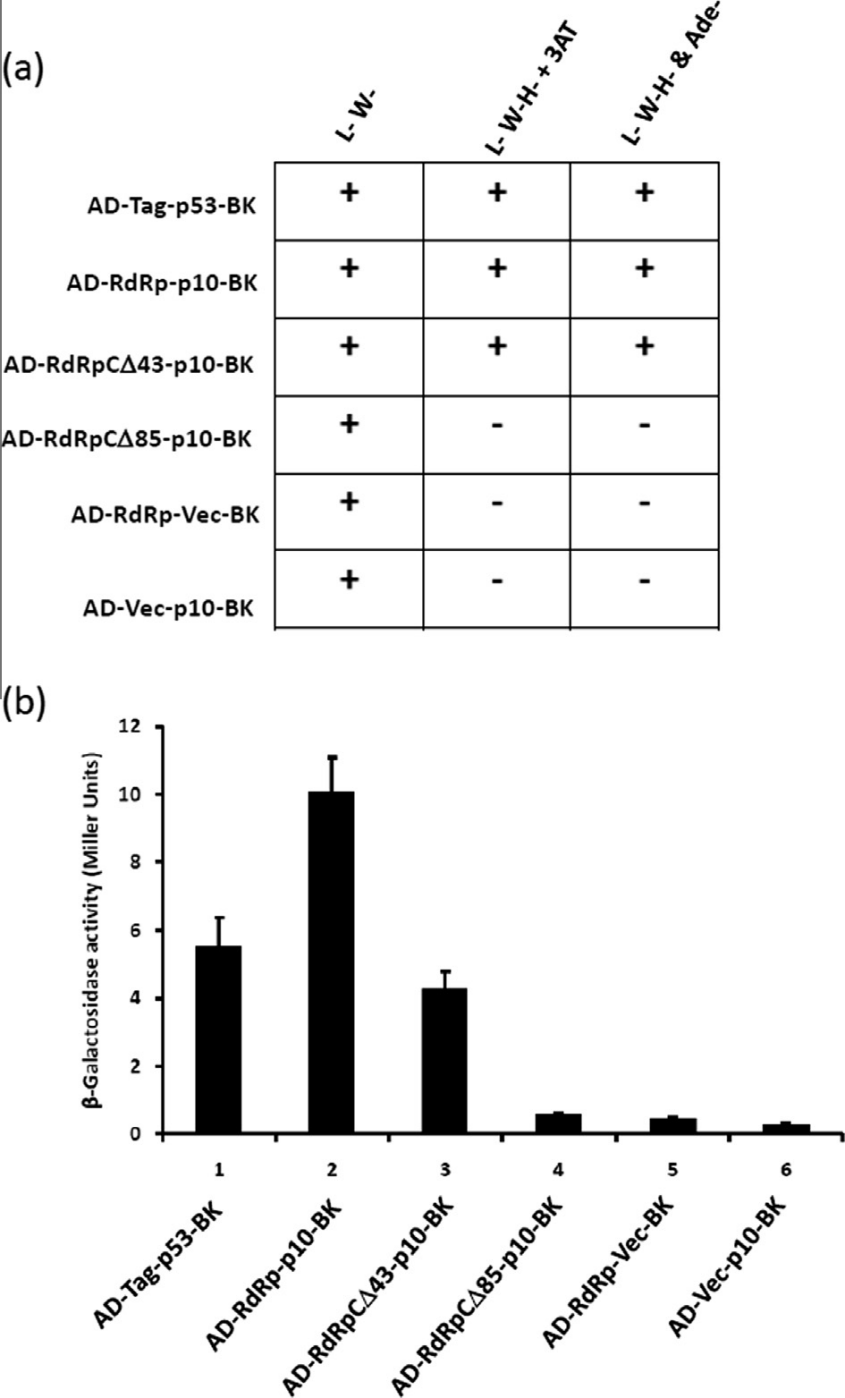
Mapping of p10 interacting region on RdRp using Y2H assay. (a) RdRp and its C-terminal deletion mutants (pGAD-RdRpCΔ43 and pGAD-RdRpCΔ85) were cotransformed with pGBK p10 and plated on L^−^ and W^−^ transformation selection plates. The colonies obtained were restreaked on fresh L^−^ & W^−^ plates (column 1). The cells grown on L^−^ & W^−^ plates were replica plated on interaction selection plates [(L^−^, W^−^ & H^−^ + 5 mM 3AT (column 2); L^−^, W^−^, H^−^ & Ade^−^ (column 3)]. (b) Quantitation of interaction by β-Galactosidase assay: The β-galactosidase assay was carried out with cells grown on L^−^, W^−^, H^−^ and Ade^−^ plates. The results obtained are presented as a bar diagram (*Y*-axis, β-galactosidase activity units; *X*-axis Y2H interactions). The pGAD-T-antigen-p53-pGBK was used as positive control and pGAD-RdRp-Vector-pGBK and pGAD-Vector-p10-pGBK were used as negative control (grown on L^−^ & W^−^ galactose media). The pGAD-RdRpCΔ85-p10-pGBK was also grown in L^−^ & W^−^ galactose media.

RdRp was subjected to foldindex analysis to predict ordered and disordered regions [24] (http://bip.weizmann.ac.il/fldbin/findex). As shown in Fig. 3 foldindex analysis indicated that the C-terminal domain (residues 37–94 from the C terminus) has propensity to be disordered (shown in red), when compared to rest of the polypeptide which is folded (shown in green). A similar foldindex analysis of RdRps from other *Sobemovirus* members indicated that the C-terminal disordered domain is conserved across the genus (Fig. S1). The N-terminal domain of RdRp is predicted to be disordered for some sobemoviruses. Intrinsically disordered proteins/regions in folded proteins acquire different conformations and are involved in protein–protein interactions [3,25,26]. In order to test whether the C-terminal disordered domain of RdRp is required for interaction with p10 domain, C-terminal deletion mutants [RdRpCΔ43 (which removes most of the folded region and few amino acids of unfolded region) and RdRpCΔ85 (which removes most of the disordered region)] were generated and cloned in pGADT7 vector. The deletion mutants of RdRp were cotransformed with pGBKT7p10 and interaction screen was carried out as described above. As shown in Fig. 4(a), the RdRpCΔ43-p10 cotransformant grew well on triple dropout plates containing 3AT and quadruple dropout plates suggesting that the interaction was not abolished with p10. However deletion of 85 residues from C-terminus of RdRp abolished the growth on these media suggesting that the C terminal disordered domain of RdRp may be involved in the interaction. Interestingly, β-galactosidase assay showed that the interaction strength could be significantly affected upon deletion of C terminal 43 residues of RdRp [Fig. 4(b) bar 3)]. These results suggest that overall conformation of C-terminal domain of RdRp may be important for interaction with p10.

**Supplementary Fig. 2:**
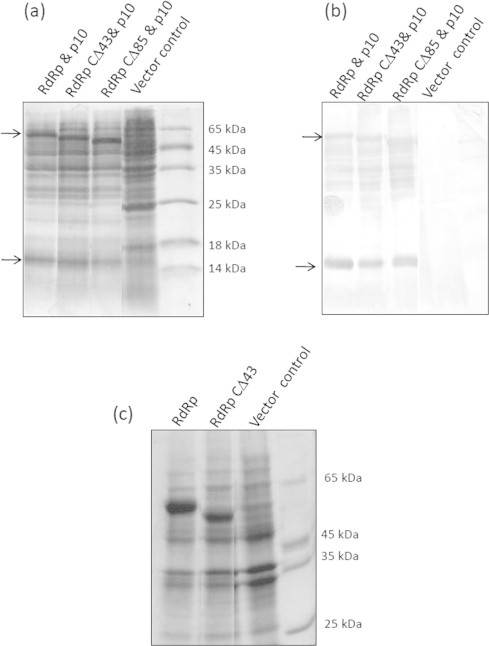
Protein expression analysis: (a) Coexpression of RdRp, RdRpCΔ43 and RdRpCΔ85 (lacks His tag) with p10 (contains N-terminal His tag). The corresponding RdRp and p10 positions were shown by arrow. (b) Western blot analysis of respective samples from (a), western blotting was carried out first with anti His tag antibody followed by same blot re-probed with RdRp antibody. Empty vector (pRSF duet) transformed cells were used as control (c) Expression and SDS–PAGE analysis of pET22b RdRp and RdRpCΔ43. Empty vector (pET22b) transformed cells were used as control.

**Fig. 5:**
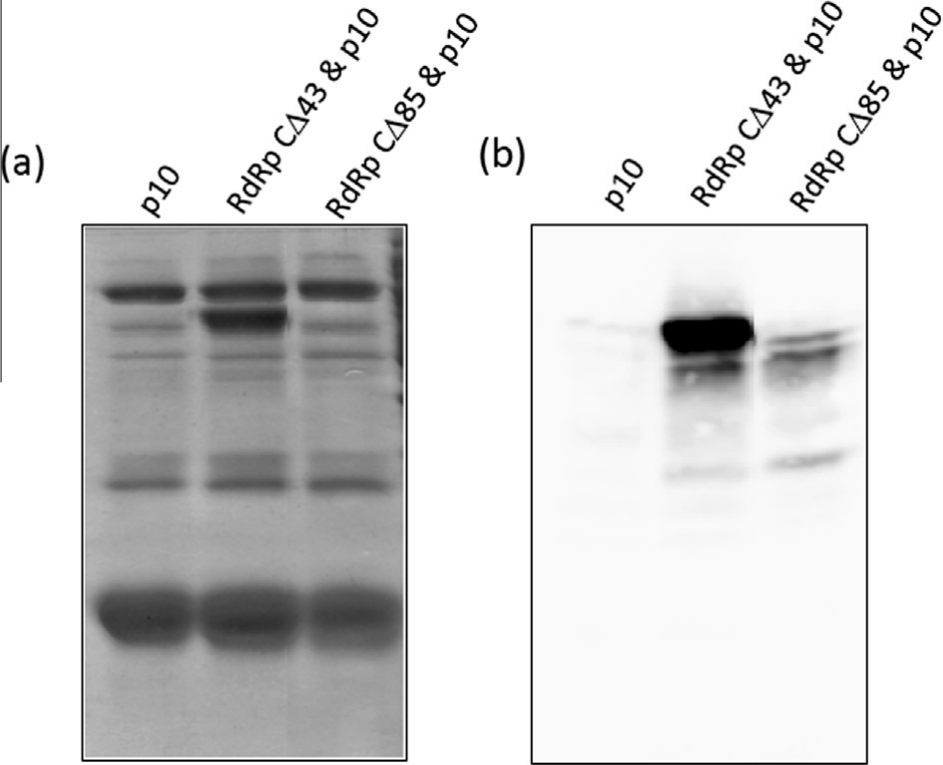
Coexpression and pull down assay of untagged RdRp C-terminal deletion mutants with 6xHis-p10: Untagged RdRp C-terminal deletion mutants were coexpressed with 6xHis-tagged p10 and pull down was carried out using Ni–NTA beads. (a) SDS–PAGE analysis of eluted proteins after pull down; lane 1 shows pull down of His-p10 alone; lanes 2 & 3 show pull down of RdRp CΔ43 and RdRp CΔ85 with His-p10 respectively. (b) Western blot analysis of respective samples from (a) with anti RdRp antibodies.

The interaction between RdRp C-terminal deletion mutants and p10 was also tested using coexpression and pull down assay methods. The PCR products of RdRp deletion mutants RdRpCΔ43, and RdRp-CΔ85 were cloned separately in pRSF Duet vector along with the p10. The pRSF Duet p10-RdRpCΔ43 and p10-RdRpCΔ85 clones were transformed into BL21(DE3)pLys-S competent cells and allowed to coexpress as described in Section 2 (Fig. S2). The cells were lysed and Ni–NTA affinity pull down was carried out as described in Section 2. The eluted fractions were analyzed by SDS–PAGE and western blotting. As shown in Fig. 5(a) lane 2 and (b) lane 2, RdRpCΔ43 mutant was copurfied along with p10, suggesting that the interaction was not abolished. However, deletion of 85 residues from C-terminus of RdRp abolished the interaction with p10 (Fig. 5(a) lane 3 and (b) lane 3), indicating involvement of C-terminal disordered domain of RdRp in interaction with p10.

### 3.4 Effect of RdRp–p10 interaction on RdRp activity

**Fig. 6:**
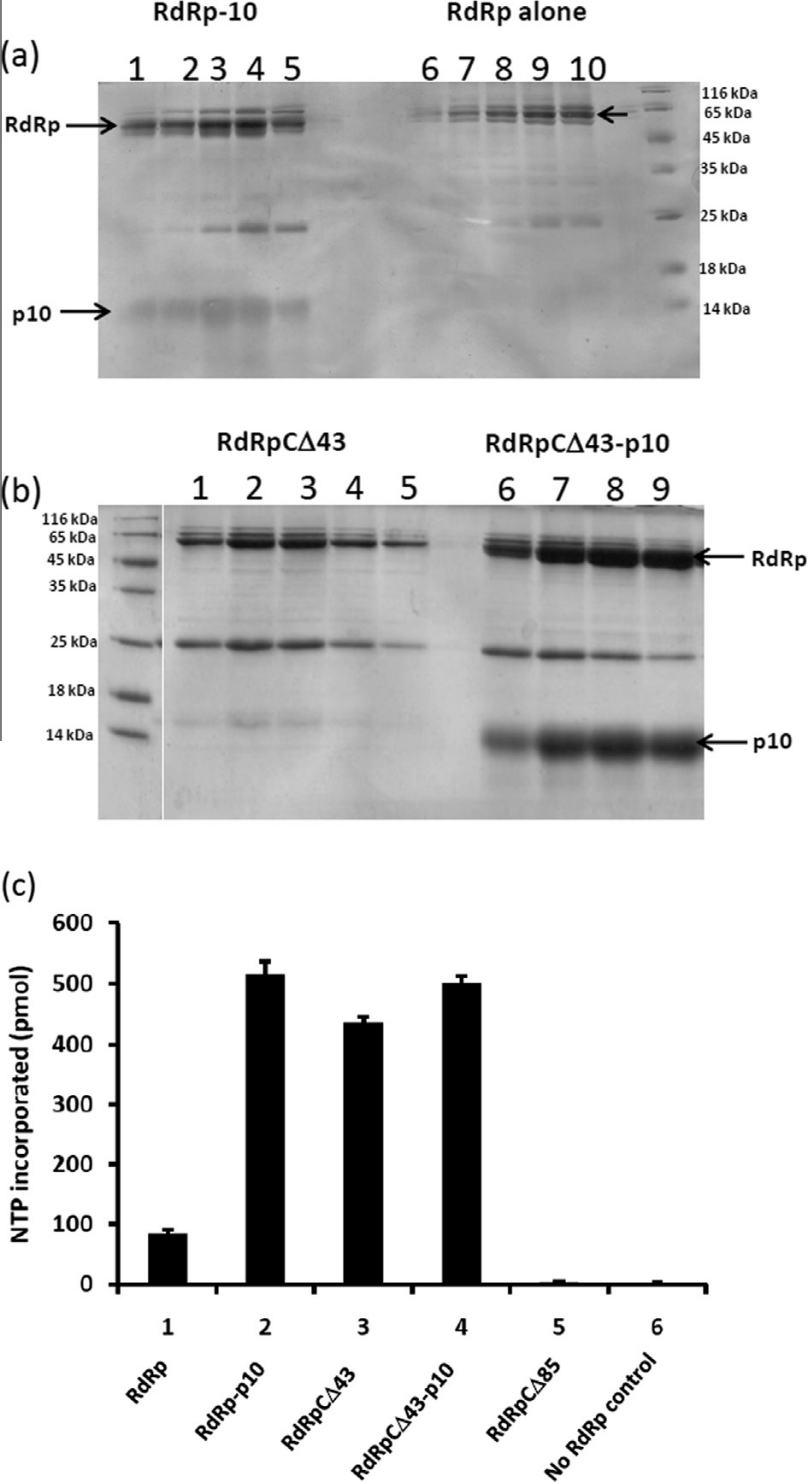
Purification of RdRp, RdRpCΔ43, RdRp-p10 complex and RdRpCΔ43-p10 complex and *in vitro* RdRp assay: Untagged RdRp and RdRp CΔ43 were coexpressed with 6xHis tagged p10 and purified by Ni–NTA chromatography. The RdRp and its mutant that formed complex with p10 was separated on SDS–PAGE and detected with coomassie brilliant blue staining. For the purification of RdRp alone and RdRpCΔ43 alone, they were cloned in pET22b vector to give C-terminal His tag. These clones were expressed and purified as described in methods section. (a) RdRp–p10 complex (lanes 1–5) and RdRp alone (lanes 6–10); (b) RdRpCΔ43 (lanes 1–5) and RdRpCΔ43–p10 complex (lanes 6–9). (c) *In vitro* RdRp assay: The reaction products were analyzed by filter binding assay, RdRp assay with RdRp alone bar 1; RdRp–p10 complex bar 2; RdRpCΔ43 bar 3; RdRpCΔ43–p10 bar 4; RdRpCΔ85 bar 5 and without RdRp control, bar 6.

To study further the importance of the interaction between RdRp and p10, RdRp-p10 and RdRpCΔ43-p10 were coexpressed (Fig. S2) and purified (Fig. 6a and b) as described in Section 2. Similarly, RdRp alone and RdRpCΔ43 alone were also independently cloned in pET22b (results in C-terminal His tag), expressed (Fig. S2) and purified in BL21 DE3 *E. coli* cells expressing chaperones as described in Section 2(Fig 6a and b). In both the cases 25 kDa and 65–70 kDa *E. coli* proteins were observed as contamination; however, identity of these proteins was not clear (Fig. 6a and b). Interestingly, the yield of RdRpCΔ43-p10 complex was about 2–3-folds higher than RdRp–p10 complex (Fig. 6a and b).

An *in vitro* RdRp assay was carried out with 2 μg each of RdRp–p10 and RdRpCΔ43–p10 complexes and compared with respective RdRp and RdRpCΔ43 alone controls. Interestingly as shown in Fig. 6(c) RdRp–p10 complex showed five to sixfold higher activity (Fig. 6(c) bar 2) when compared to RdRp alone (Fig. 6(c) bar 1), suggesting p10 might positively regulate RdRp activity. However, such activation of RdRp activity was also observed when C-terminal 43 residues of RdRp were deleted (Fig. 6(c) bar 3), indicating that these residues are not essential for polymerase activity and negatively regulate RdRp activity. The RdRpCΔ43–p10 complex showed activity similar to that of RdRpCΔ43 alone (Fig. 6(c) bar 4). However, RdRpCΔ85 failed to interact with p10 and was also enzymatically inactive (Fig. 6(c) bar 5). These results suggest p10 may interact and alter the conformation of RdRp C-terminus thereby activate RNA synthesis.

## 4 Discussion

Identification of viral replication proteins is an important step towards the understanding of mechanism of replication. In the present study Y2H interaction studies were carried out to identify SeMV encoded proteins that might interact with RdRp. The results showed that RdRp interacts moderately with pro and strongly with p10 domain of polyprotein 2a (Figs. 1 and 2). SeMV RdRp did not show detectable interaction with VPg, p8 (Figs. 1 and 2) or CP although it was expected that RdRp might interact with VPg, as VPg acts as primer during initiation of RNA synthesis. Lack of detectable interaction between VPg and RdRp may be because either these interactions are transient or the assembly of replicase complex is required for such interactions. The later possibility is substantiated by the observation that VPg could not be nucleotidylylated by recombinant RdRp [18].

Assembly of replicase on the surface of membrane is critical for *in vivo* replication. The TYMV protease domain of 140 K protein was shown to interact with 66 K polymerase and help in assembly of replication complex at the periphery of chloroplasts [27]. The potyvirus proteinase precursor 6 K/NIa protein and NIb (polymerase) interaction was shown to be important for recruitment of polymerase to the replication initiation complexes [28–30]. The N-terminus of SeMV pro consists a transmembrane domain and is important for targeting polyproteins to the site of replication [19]. It is therefore, possible that SeMV pro may assist in the recruitment of RdRp to the site of replication and or assembly of replication complex via its interaction with RdRp. However, in the Y2H assays presented in this paper, a part of the membrane anchor domain (1–132 residues) was removed from the protease domain. In spite of this, RdRp exhibited moderate interaction with the NΔ70 protease domain.

The C-terminal amino acid sequences of *Sobemovirus* RdRps are not conserved and are predicted to be disordered (Figs. 3 and S1). Such disordered domains play an important role in mediating protein–protein interactions and modulating the function of other interacting partners [3,25,26]. Natively unfolded VPg of SeMV was shown to interact with pro in *cis* and activate the protease [31]. Similarly disordered p8 domain was shown to interact with p10 domain and enhance the ATPase activity [21]. However, deletion of C-terminal 43 residues did not result in loss of interaction, although interaction strength was affected significantly (Figs. 4 and 5(a) and (b)). It is therefore, possible that overall conformation of C-terminal domain of RdRp is important for optimal interaction with p10. On the other hand, deletion of 85 residues from the C-terminus resulted in complete loss of interaction with p10 (Figs. 4 and 5(a) and (b)). Interestingly, when RdRp was coexpressed with p10, it copurified with p10 demonstrating further that RdRp interacts with p10 (Fig. 6) and such an interaction leads to 5–6-fold increase in the polymerase activity as compared to the activity of RdRp alone (Fig. 6c). Further, deletion of 43 residues from the C terminus of RdRp also led to a similar fold increase in activity of RdRp (Fig. 6c). These results suggest that p10 acts as a positive regulator of the RdRp activity via its interaction with the C terminal domain in the absence of which the activity would be highly reduced. One possible explanation for enhanced activity upon deletion of C-terminal residues could be due to improved stability of the CΔ43 RdRp or CΔ43 RdRp–p10 complex when compared to full length RdRp alone. It was shown that deletion of C-terminal 21 residues from the C-terminus (consists a transmembrane helix) of HCV NS5B resulted in improved stability and activity, probably because of reduction in the aggregation induced by C-terminal hydrophobic helix [32,33]. It is possible that the conformation of the C-terminal domain of RdRp is stabilized upon interaction with p10 which results in enhanced polymerase activity of RdRp–p10 complex.

The p10 domain of SeMV polyprotein 2a is poorly studied due to lack of similarity with the known proteins. Recent studies have suggested that it could be an ATPase and its activity could be stimulated by p8 domain in *cis* [21]. The p10 was also shown to interact with MP and it was speculated to be involved in movement [34]. Present study highlights p10 as a positive regulator of RdRp. The *in silico* analysis of p10 suggested that it possesses random coils at N and C terminus and an alpha helix in the middle. The random coils, like disordered proteins, undergo conformational changes and acquire secondary structure upon protein–protein interaction [35]. It could therefore possible that p10 may acquire different conformation upon interaction with different proteins and thereby modulate multiple functions including replication, movement and other yet to be identified functions.

In conclusion, the ancillary proteins that interact with SeMV RdRp were identified for the first time. P10 interacted strongly with RdRp and positively modulated the RdRp activity probably by stabilizing the C-terminal domain of RdRp. However precise *in vivo* role of RdRp–p10 interaction remains to be elucidated in future.

## Figures and Tables

**Table 1:** Oligonucleotides used in this study.

Name	Sequence (5′–3′)	Description
ΔN70pro Off Fw	5′ CGCACATATGGAGGCAAAGCAGGACAGAGTC 3′	Used for PCR amplification of ΔN70pro and cloning in pGBKT7 vector. *NdeI* restriction site underlined
ΔN70pro Rev	5′ CGGGATCCTTACTCATTAGACCTTAAGAGG 3′	Used for PCR amplification of ΔN70pro and cloning in pGBKT7 vector. The BamHI site underlined
VPg off Fw	5′ CCCA**GCTAGC**CATATGACTCTCCCACCGGAGC 3′	Used for PCR amplification of VPg and cloning in pGBKT7 vector. *NheI* and *NdeI* sites are shown bold and underlined respectively
VPg Rev	5′ CGGGATCCTCACTCTTGAGCGTTTTCCC 3′	Used for PCR amplification of VPg and cloning in pGBKT7 vector. The *HamHI* site underlined
p10 Fw1	5′ GCCC**GAATTC**CACCGTCGCTGTTGAGAAT 3′	Used for PCR amplification of p10 and cloning in pRSF duet vector. *EcoRI* is shown in bolt
p10 Fw2	CTAGCTAGC**CATATG**ACCGTCGCTGTTGAG	Used for PCR amplification of p10 and cloning into pGBK vector. NdeI site is shown in bolt
p10 Rev1	5′ CCCGTCGACTTATTCCTGCTTGTAATAACAAGG 3′	Used for PCR amplification of p10 and cloning in pRSF duet vector. *SalI* site is underlined
p10 Rev2	5′ CGGGATCCTCATTCCTGCTTGTAATAACAAGG 3′	Used for PCR amplification of p10 and cloning in pGBKT7 vector. *BamHI* site is underlined
p8 Fw	5′ CTAGCTAGCCATATGAGTTTAATCCTTCCAGAGTCC 3′	Used for PCR amplification of p8 cloning in pGBKT7 vector. The *NdeI* restriction site is underlined
p8 Rev	5′ CGGGATCCTCAGTAACACAGAGAGCAACAAG 3′	Used for PCR amplification of p8 and cloning in pGBKT7 vector. The *BamHI* site underlined
RdRp Tsen Off	5′ GCGT**GCTAGC**CATATGACCGTCGCTGTTGAGAATTTTAAACTGCCAGC 3′	Used for PCR amplification of RdRp and cloning in pGADT7 and pRSF duet vectors. *NheI* and *NdeI* sites are shown in bold letters and underlined respectively
RdRp Rev	5′ CGCCTCGAGCGAATCCGCACCATAGCACCCTG AGCA 3′	Used for PCR amplification of full length RdRp and cloning in pGADT7 and pRSF duet vectors. *XhoI* restriction site was underlined
RdRp CΔ43 Rev	5′ TTACTCGAGGTCTCCAGAGTGTTCGC 3′	Used for PCR amplification of RdRp C-terminal 43 amino acids deletion mutant and cloning in pGADT7 and pRSF duet vector. *XhoI* restriction site underlined
RdRp CΔ 85 Rev	5′ GGGTTACTCGAGAGGCCAGTGGGGCGAA G TTTC 3′	Used for PCR amplification of RdRp C-terminal 85 amino acids deletion mutant and cloning in pGADT7 and pRSF duet vector. XhoI restriction site underlined
CP off Fw	5′ GCCCCATATGGCGAAAAGGCTTTCGAAACAACAG 3′	Used for PCR amplification of CP. *NdeI* restriction site underlined
CP ORF Rev	5′ TCA CCC GGG GTT GTT CAG GGC TGA GGC AG 3′	Used for PCR amplification of CP. SmaI restriction site underlined
